# Mining Biological Pathways Using WikiPathways Web Services

**DOI:** 10.1371/journal.pone.0006447

**Published:** 2009-07-30

**Authors:** Thomas Kelder, Alexander R. Pico, Kristina Hanspers, Martijn P. van Iersel, Chris Evelo, Bruce R. Conklin

**Affiliations:** 1 Gladstone Institute of Cardiovascular Disease, San Francisco, California, United States of America; 2 Departments of Medicine, and Molecular and Cellular Pharmacology, University of California San Francisco, San Francisco, California, United States of America; 3 Department of Bioinformatics - BiGCaT, Maastricht University, Maastricht, The Netherlands; University of the Western Cape, South Africa

## Abstract

WikiPathways is a platform for creating, updating, and sharing biological pathways [1]. Pathways can be edited and downloaded using the wiki-style website. Here we present a SOAP web service that provides programmatic access to WikiPathways that is complementary to the website. We describe the functionality that this web service offers and discuss several use cases in detail. Exposing WikiPathways through a web service opens up new ways of utilizing pathway information and assisting the community curation process.

## Introduction

WikiPathways is an online resource for biological pathway information and a platform for community-based curation [1]. Currently, WikiPathways contains more than 600 pathways, representing various species from bacteria and fungi to plants and animals. New species are being added on demand, and pathways are being created and edited almost daily. These pathways combine different types of biological knowledge, including protein interactions, metabolic reactions, annotations from gene, protein and metabolite databases and references to scientific literature. Using a Java-based pathway editor, researchers can create, edit and annotate pathways directly on the website [2].

**Figure 1:**
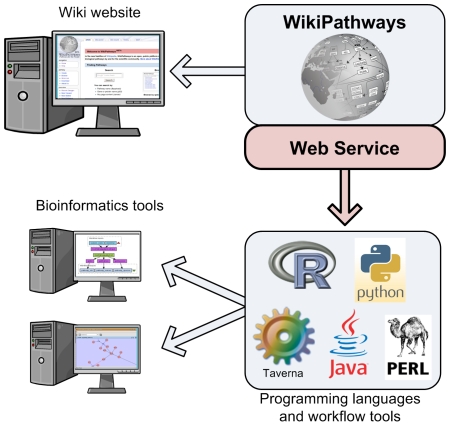
WikiPathways can be accessed by end-users from the wiki-style website. In addition, the WikiPathways web service provides a programmatic interface that can be used in many programming languages, including R, python, Java and perl and in workflow tools such as Taverna. Using this interface, new pathway analysis tools can be built and existing bioinformatics tools can be extended with pathway-based functionality.

WikiPathways is accessed by the biology community mainly via a wiki-style website. In addition to the website, we recently implemented a web service that provides programmatic access to WikiPathways (figure 1). This makes it easier to integrate biological pathways in existing applications and provides a framework for pathway-centered analysis of experimental data. In contrast to other pathway resources with managed curation teams (e.g., KEGG [3] and Reactome [4]) or focused on distributing and querying pathway information (e.g., Pathway Commons [5]), WikiPathways is a primary source for richly annotated pathway content open to community curation. With the addition of web services, users have expanded access and advanced methods that uniquely apply to WikiPathways content. In this article, we discuss the web service functionality in more detail and highlight several use cases.

Supplementary data, including full documentation, example client implementations, and source code, are available at http://www.wikipathways.org/webservice. The source code of the web service and all examples are licensed under the Apache 2.0 open-source license.

## Results and Discussion

### Web service functionality

The web service provides an interface to WikiPathways that can be accessed through the Simple Object Access protocol (SOAP), and the data structure and available functions are described in the Web Service Description Language (WSDL). Both SOAP and WSDL are widely supported standards. The web service provides access to all pathway information on WikiPathways in several different forms. Complete pathways can be downloaded in XML format (GPML) or a plain text file listing the biological entities and their identifiers. Image versions of a pathway can be retrieved in several graphics formats, including rasterized formats (e.g., Portable Network Graphics (PNG)) and vector graphics formats (e.g., Scalable Vector Graphics (SVG) and Portable Document Format (PDF)). Additionally, color information can be specified to highlight specific elements of the pathway (e.g., to color protein entities according to their measured expression). Individual interactions between biological entities that are defined in the pathways can be retrieved separately. Furthermore, information related to community-based curation, such as revision history and recently edited pathways, can be queried. A full list of available functions can be found in the supplementary data.

An index of all pathways is maintained using the Apache Lucene library [6]. This feature makes it possible to perform advanced search queries through the web service. All textual information on the pathway is included in the index to allow for keyword searches. In addition, the index uses synonym databases [2] to cross-reference between various biological databases and the entities on the pathways. This allows for queries to find all pathways for any given biological identifier, regardless of the identifier system that is used to annotate the pathway. For example, when the query is an Affymetrix probeset identifier, all pathways containing genes that map to that probeset will be returned.

The web service also allows client software to publish information to WikiPathways. New pathways can be uploaded and pathways can be modified or labeled according to quality standards. This enables scripts to perform quality monitoring and notification to assist the manual community-based curation process. This concept has already been successfully applied to other wiki's, such as Wikipedia [7]. To prevent scripts from systematically overwriting pathways with invalid data, write access to the web service is restricted to a subset of user accounts. Users who require write access for their script can request it from the WikiPathways site administrators.

To assist programmers in building applications that use the WikiPathways web service, several toolkits and programming libraries exist. Libraries to handle SOAP requests and responses are available for practically any programming language. Additionally, several bioinformatics tools, such as Taverna and GenePattern, support plugging in SOAP web services by writing only little or no extra code. This makes it easy to integrate WikiPathways in existing pipelines. To facilitate working with the GPML pathway format, we maintain an open-source Java library that provides a high-level API to process GPML. This library contains methods to read and write pathways in several file formats and to modify information in the pathway. Furthermore, it provides an object-oriented interface to the WikiPathways web service, including support for caching downloaded pathway information locally to improve performance. For each described use-case, example code is provided that demonstrates the use of available toolkits and libraries. The supplementary data include a list of useful libraries in several programming languages.

### Web applications

The web service can be used to build web applications that provide end-users access to specific WikiPathways functionality. Research groups can build a website that queries, processes and presents information from WikiPathways in a fully customized way. As an example, we implemented two web applications, each highlighting a unique functionality of the web service. The first application is an improved search application with more advanced functionality, available at http://search.wikipathways.org. The default WikiPathways search is based on a Google Custom Search Engine and allows searching through all the text on pathway pages, but doesn't use the biological context that is defined in the GPML format. The improved search application uses the web service functions that query the Lucene index and allows users to perform searches with biological context, such as using biological identifiers and filtering by species. Furthermore, the results are presented as thumbnail images, making it easier to choose the relevant result.

**Figure 2:**
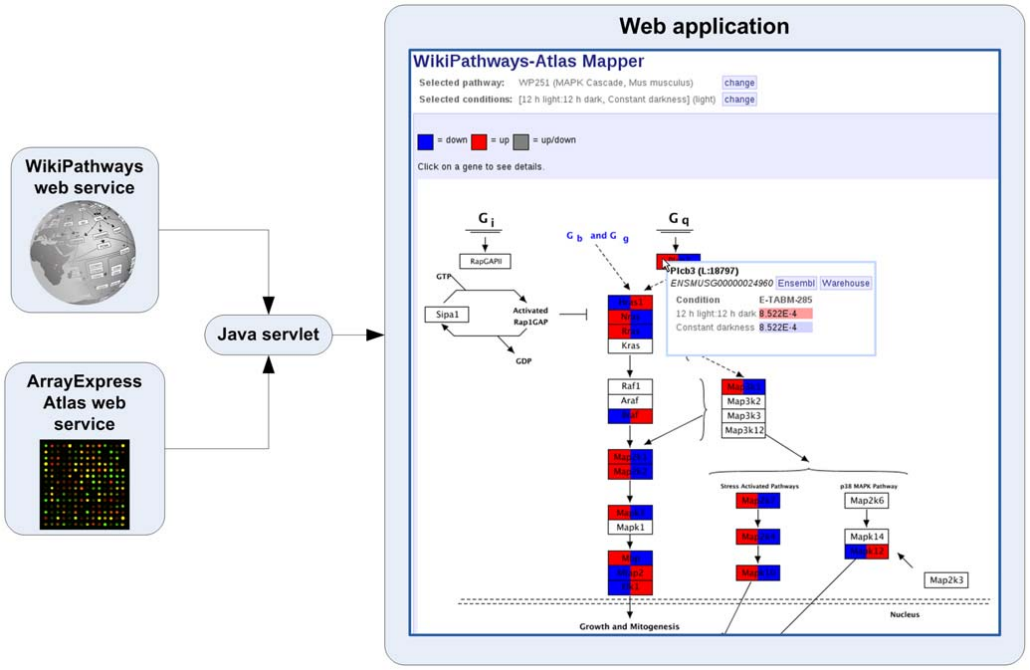
Web application that integrates pathways with gene expression information. Pathway and gene expression information are retrieved from the WikiPathways and ArrayExpress Atlas web services respectively. A Java servlet integrates this information and publishes it to an interactive web application. In this web application, users can view the information on an interactive pathway diagram.

The second example is a web application that demonstrates integration of pathway information with other types of data. This application visualizes gene expression information from ArrayExpress Atlas 8 on a WikiPathways pathway. ArrayExpress Atlas is a curated set of gene expression datasets that are publicly available. In this example, the user can specify a pathway from WikiPathways and a set of experimental conditions defined in ArrayExpress Atlas. First, all gene identifiers on the pathway will be mapped to Ensembl using the synonym database. The resulting Ensembl identifiers are passed to the ArrayExpress Atlas web service, which returns the corresponding experiments and p-values for the differentially expressed genes. Second, the WikiPathways web service will be used to download a colored version of the pathway image that will be displayed to the user (figure 2). This application can be used to get a quick overview of how known pathway interactions relate to observed gene expression in a given experimental condition and is available at http://atlas.wikipathways.org. Currently, 716 of the 1000 experiments on ArrayExpress Atlas can be mapped onto at least one of the WikiPathways pathways, representing 9357 unique genes affected in one or more experiments across 7 different species.

Research groups are encouraged to build their own client-side web applications based on the WikiPathways web service and our open source libraries. This could include applications that present WikiPathways content in a customized way or integrate pathways with other data. For example, research groups focused on metabolic pathways could create an application that presents the pathways in combination with detailed enzymatic information, while the genetics community could create a web application that combines polymorphism information with the genes from a pathway.

### Assisting community-based curation

The biology community manually moderates the content on WikiPathways. Being able to quickly respond to mistakes as well as acts of vandalism is an important aspect of community-based curation [9]. Many types of erroneous data can be identified automatically. For example, wrongly annotated gene, protein, or metabolite entities are identified by a simple computer script. The web service enables us to create “bots” that continually watch the pathway content for errors and notify curators when incorrect information is introduced. Currently, various types of bots run on a daily basis and check for incorrect database annotations, missing literature references, or incorrectly defined interactions. Each bot generates an HTML report that provides statistics and an overview of pathways that need to be corrected. By monitoring these reports over time, we can assess the effectiveness of community-based curation.

Community involvement in pathway curation on WikiPathways could be further stimulated by using the web service to build applets that can be included in any webpage or desktop. These applets could display user-specific information, such as recent changes on pathways that have been edited by the user, recent discussion items, or listing other users that are interested in similar pathways. Users could install this applet on their own webpage or on their desktop, for example using Google Gadget interface [10].

### Querying interactions in Cytoscape

WikiPathways includes interactions between biological entities that can be represented as interaction networks. For example, metabolic reactions or protein activation events that are defined in a pathway can be viewed as binary interactions that form a graph. This enables network-based analysis [11] and integration with various datasets, such as protein-protein interactions [12], [13]. Cytoscape [14] is a widely used tool for visualization and analysis of biological networks. The core Cytoscape functionality can be extended by external developers via a plug-in mechanism. We used this mechanism to build a plug-in that allows Cytoscape to work with GPML pathways [2]. We recently extended the plug-in with functionality from the WikiPathways web service. This new functionality makes it possible to search for pathways on WikiPathways directly from within Cytoscape, and these pathways can be loaded as an interaction network and then analyzed and integrated with experimental data [15]. Furthermore, existing networks in Cytoscape can be expanded with interactions defined in WikiPathways. Network analysis of biological pathways can also be a useful tool to augment pathways with knowledge from various resources. For example, cross-talk between biological pathways can be identified by projecting pathways on large protein-protein interaction networks [16]. The Cytoscape plug-in is a typical example of how the web service can be used to enrich external analysis tools with pathway content.

Future versions of WikiPathways will allow the user to define the semantic meaning for each interaction in a pathway. This can be used to improve the web service with functions that query and filter interactions based on this semantic information. For example, queries, such as “show me all proteins that inhibit phosphorylation of protein X” could be performed. Including semantics also opens new possibilities for analysis in Cytoscape, for example the signaling pathway impact factor analysis method [17], which can use the distinction between activation or inhibition interactions.

### Integration in bioinformatics workflows

A primary difficulty in bioinformatics is integrating erratically formatted data from different resources [17], [18]. The development of web services for biological databases makes this easier and enables usage of workflow tools, such as Taverna [19]. Taverna provides a framework for creating workflows that integrate and process different types of data. For instance, a workflow could be used to integrate microarray experiment data with quantitative trait loci to filter for relevant genes [20]. Knowledge represented as a pathway is especially suited for integration with other biological data, since it typically covers multiple levels of the biological information hierarchy. Transcriptomics, proteomics, or metabolomics data can be integrated with pathway information to aid in the understanding of the underlying biological mechanisms. We created several Taverna workflows based on the WikiPathways web service, such as a workflow that finds relevant pathways based on a list of over-expressed genes, proteins, or metabolites. These workflows can be downloaded from http://www.myexperiment.org/packs/30. Other possible workflows that could be implemented in Taverna include cross-species comparisons of pathway information, or batch visualization of expression data.

The WikiPathways web service could be used as framework for building data analysis tools that make use of pathway information. Pathways can be used for visualizing experimental data in a biological context [21], finding relevant biological mechanisms and improving statistical power of an experimental analysis [22], [23]. The WikiPathways web service provides all of the functionality necessary to perform these methods. Pathways can be downloaded as images where the pathway components can be given user defined colors, allowing for experimental data analysis. Genes, proteins and metabolites are linked to several identifier systems, providing information to perform enrichment analyses.

### Integration with online databases

Pathways typically consist of different types of biological entities, such as genes, proteins and metabolites. For each entity type, different biological databases are available, and each presents unique information about the entities in a different way. With the WikiPathways web service, we aim to encourage database developers to integrate pathway information into their online data presentation to provide more biological context. WikiPathways links pathway components to over 50 supported biological databases, including Entrez Gene, Ensembl, Affymetrix, ZFIN, TAIR, and ChEBI. Tools that use information from any of these databases can use the web service to retrieve relevant pathway information per biological entity. For example, the list of pathways that contain a given gene identified by an Ensembl ID could be retrieved and displayed on the Ensembl web page for that gene or any website indexed by Ensembl identifiers. The website could display the pathway name, URL and/or thumbnail image of the pathway. A similar approach could be taken by metabolic databases (e.g., PubChem, ChEBI, or ChemSpider), protein databases (e.g., UniProt), model organism databases (e.g., MGI, ZFIN, WormBase, FlyBase, or TAIR), measurement platforms (e.g., Affymetrix, Illumina, or Agilent), or even literature databases, such as PubMed.

Future versions of WikiPathways will support the export of pathways in BioPAX format. This will make it easier to integrate WikiPathways with other pathway databases and resources, such as PathwayCommons. This functionality will be available in the web service, so that integrated pathway resources can easily keep the pathway information from WikiPathways up-to-date.

## Conclusions

The WikiPathways web service provides an interface for programmatic access to community-curated pathway information. It provides a flexible framework for building or extending tools that use pathway information from WikiPathways. The web service can be used by software developers to build or extend tools for analysis and integration of pathways, interaction networks and experimental data. The web services are also useful for assisting and monitoring the community-based curation process. By providing this web service, we hope to help researchers and developers build tools for pathway-based research and data analysis.
